# A Simple Restriction, a Complex Collapse: Multisystem Wernicke Encephalopathy Triggered by Self-Starvation

**DOI:** 10.7759/cureus.96673

**Published:** 2025-11-12

**Authors:** Mohammadreza Akbarian Khorasgani, Melika Khalifeh Hadi, Pouriya Katouzi, Chalachew Tiruneh

**Affiliations:** 1 Department of Neurology, Qilu Hospital of Shandong University, Jinan, CHN; 2 Department of Anatomy and Neurobiology, Shandong University, Jinan, CHN

**Keywords:** body image distortion, eating behavior, interdisciplinary case report, malnutrition, non-alcoholic encephalopathy, self-starvation, thiamine deficiency, wernicke encephalopathy

## Abstract

Wernicke encephalopathy (WE) is an acute neuropsychiatric emergency caused by thiamine deficiency, most commonly associated with chronic alcoholism but increasingly recognized in non-alcoholic contexts. This report describes a 23-year-old woman who developed WE secondary to prolonged self-imposed starvation driven by distorted body image. She had deliberately restricted food intake for several weeks, believing she was overweight, which led to severe malnutrition, weight loss, and profound weakness. On admission, she presented with bilateral ptosis, nystagmus, and lower limb weakness but without overt confusion. Laboratory evaluation revealed severe normocytic anemia, hypoalbuminemia, hypophosphatemia, hypomagnesemia, and critically low vitamin D levels. Magnetic resonance imaging of the brain demonstrated symmetric hyperintense lesions in the medial thalamus and periaqueductal gray matter, findings characteristic of WE. High-dose intramuscular thiamine was initiated immediately, alongside electrolyte correction, nutritional rehabilitation, and transfusion for anemia. The patient’s neurological symptoms and biochemical abnormalities improved steadily, and she was discharged in stable condition after nine days of hospitalization. This case underscores that WE can occur in young, non-alcoholic individuals with self-starvation or restrictive eating behaviors, even in the absence of a diagnosed eating disorder. The presentation may be incomplete and easily overlooked, emphasizing the importance of maintaining clinical suspicion in any malnourished or vomiting patient. Early administration of thiamine remains the cornerstone of treatment and can lead to full neurological recovery when implemented promptly. This case further highlights the need for heightened awareness of nutritional brain injury associated with modern body-image distortion and restrictive dieting practices among young adults.

## Introduction

Wernicke encephalopathy (WE) is a rapidly progressive and potentially fatal neuro-metabolic disorder resulting from thiamine (vitamin B₁) deficiency. It classically manifests with the triad of ocular disturbances, gait ataxia, and mental confusion, yet fewer than one in five patients present with the complete triad, leading to frequent misdiagnosis or delayed recognition [1]. Although WE has long been considered a complication of chronic alcoholism, mounting evidence shows that the majority of recent cases arise in non-alcoholic settings such as starvation, hyperemesis, malignancy, bariatric surgery, and eating disorders [2].

Thiamine is an indispensable cofactor for enzymes in cerebral glucose metabolism, and its depletion rapidly disrupts neuronal energy homeostasis. The resulting mitochondrial dysfunction and lactic acidosis selectively injure periventricular and diencephalic regions, particularly the medial thalamus, periaqueductal gray matter, and mammillary bodies, producing the characteristic neuroimaging pattern of WE [3]. Magnetic resonance imaging (MRI) remains the most reliable non-invasive diagnostic tool, revealing symmetric lesions in these regions that correlate closely with clinical severity.

In recent years, non-alcoholic WE has emerged as an under-recognized neurological emergency in young women who deliberately restrict nutrition for weight control. Cases linked to eating disorders or body-image distortion illustrate a tragic paradox: the pursuit of thinness in otherwise healthy individuals may culminate in catastrophic vitamin deficiency and irreversible brain injury. Despite widespread public awareness of eating-disorder-related malnutrition, the neurological consequences of self-starvation are rarely appreciated by non-specialists, and early thiamine supplementation is often overlooked.

This report describes a 23-year-old woman who, driven by a persistent belief that she was overweight, drastically reduced her food intake and subsequently developed severe malnutrition, electrolyte imbalance, and WE. Her case highlights the growing intersection between nutritional psychiatry and neurology, underscores the diagnostic value of MRI in atypical presentations, and raises an urgent question: how can clinicians identify and prevent thiamine deficiency in non-alcoholic patients whose self-imposed dietary restriction masks a life-threatening disease?

## Case presentation

A 23-year-old woman from China was admitted to the Department of Neurology at Shandong University Qilu Hospital on October 11, 2025, with a six-day history of progressive bilateral eyelid drooping, generalized weakness, and gait instability. She denied any history of alcohol use, diabetes, or psychiatric disease. Over the previous month, she had deliberately and severely restricted her food intake because of a persistent belief that she was overweight. This self-imposed dietary restriction led to profound anorexia, early satiety, and an estimated weight loss of approximately 4-5 kg. In the three weeks before admission, she developed worsening fatigue, recurrent post-prandial vomiting, and episodes of light-headedness. There was no exposure to drugs, toxins, or excessive exercise.

On admission, she appeared emaciated and pale, with a body mass index of 16.1 kg/m². Her vital signs were stable: temperature 37.1 °C, pulse 108 beats/min, respiratory rate 23 breaths/min, and blood pressure 100/64 mmHg. She was conscious and oriented but exhibited slight apathy. Neurological examination revealed equal, reactive pupils, horizontal nystagmus in both eyes, and restricted downward gaze, producing intermittent diplopia. There was mild symmetric weakness of the upper limbs (Medical Research Council grade 4/5) and asymmetric weakness of the lower limbs (right 4/5, left 3/5), accompanied by diminished deep-tendon reflexes. Bilateral Babinski signs were positive. Neck stiffness and cerebellar ataxia were absent, and sensation was intact. Systemic examination demonstrated mild hepatomegaly and a soft, non-tender abdomen without edema. Initial laboratory investigations are summarized in Table 1.

**Table 1:** Key laboratory findings on admission

Parameter	Result	Reference Range	Interpretation/Comment
Hemoglobin	45 g/L	115–150 g/L	Severe normocytic anemia
Hematocrit	13.6 %	36–45 %	Low
Red blood cell count	1.53 × 10¹²/L	3.8–5.1 × 10¹²/L	Low
Serum albumin	30.0 g/L	40–55 g/L	Hypoalbuminemia
Total protein	46.5 g/L	60–80 g/L	Hypoproteinemia
Calcium	2.05 mmol/L	2.20–2.60 mmol/L	Hypocalcemia
Phosphate	0.43 mmol/L	0.60–1.60 mmol/L	Hypophosphatemia
Magnesium	0.60 mmol/L	0.65–1.10 mmol/L	Hypomagnesemia
Vitamin B₁₂	Elevated	160–950 pmol/L	Increased (from supplementation)
Folate	1.36 nmol/L	7.0–45.0 nmol/L	Markedly decreased
25-Hydroxy Vitamin D	< 3 ng/mL	30–100 ng/mL	Critically deficient
ALT	60 U/L	< 40 U/L	Mildly elevated
AST	73 U/L	< 40 U/L	Mildly elevated
24-h Urinary sodium	Increased	—	Suggestive of renal electrolyte loss
24-h Urinary chloride	Increased	—	Suggestive of renal electrolyte loss
24-h Urinary calcium	Decreased	—	Consistent with malnutrition
24-h Urinary phosphate	Decreased	—	Consistent with malnutrition
Coagulation/Inflammatory markers	Normal	—	Within normal limits

**Figure 1:**
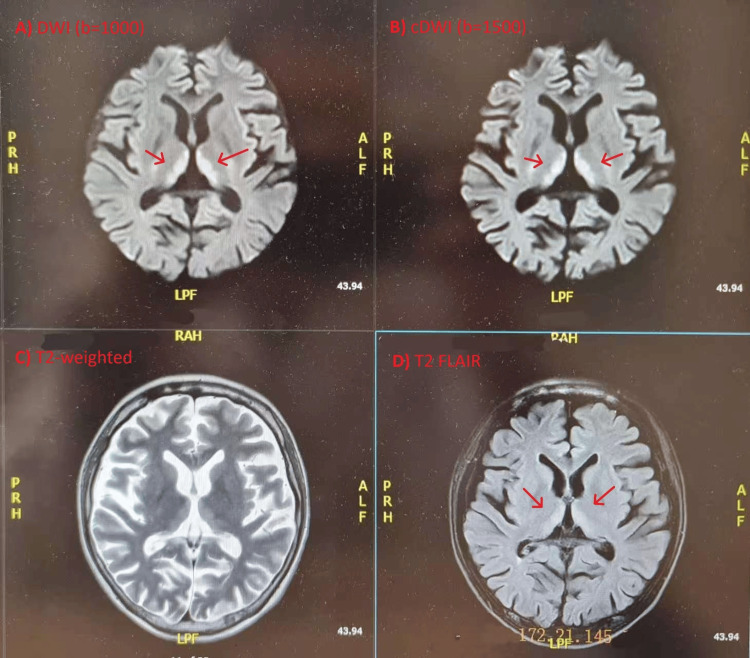
Brain MRI Axial brain MRI shows symmetric hyperintensities around the periaqueductal gray matter and medial thalami on FLAIR and diffusion-weighted imaging, consistent with Wernicke encephalopathy. FLAIR: Fluid Attenuated Inversion Recovery

**Figure 2:**
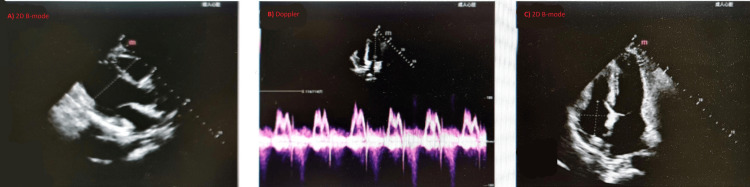
Transthoracic Echocardiography Transthoracic echocardiography revealed a normal cardiac chamber size and anatomy, mild mitral and tricuspid regurgitation, and a small pericardial effusion.

**Figure 3:**
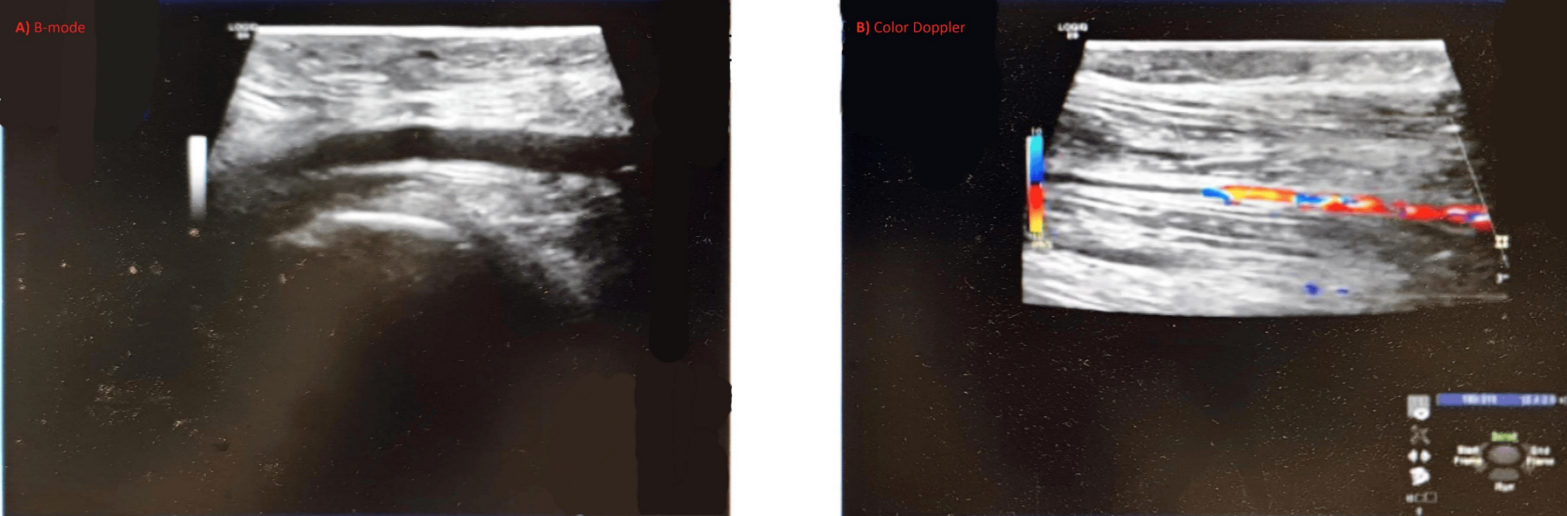
Lower-Limb Venous Doppler Ultrasound Bilateral lower-limb venous Doppler ultrasound showed patent deep veins with no evidence of thrombosis.

Cranial magnetic-resonance imaging performed two days before admission revealed symmetric T2- and fluid attenuated inversion recovery (FLAIR)-hyperintense lesions in the medial thalamus and periaqueductal gray matter with corresponding diffusion-weighted hyperintensity findings characteristic of WE (Figure 1) [4]. Abdominal computed tomography showed marked hepatic steatosis, mild hepatomegaly, periportal lymphadenopathy, gallbladder sludge, and trace bilateral pleural effusions. Echocardiography demonstrated preserved ventricular function with mild mitral and tricuspid regurgitation and a small pericardial effusion (Figure 2). Bilateral lower-limb venous Doppler ultrasound excluded deep-vein thrombosis (Figure 3). Nutritional risk screening (NRS-2002 score = 3) indicated clinically significant malnutrition requiring specialized nutritional therapy.

The patient was treated immediately with high-dose parenteral thiamine, initiated at 100 mg intramuscularly three times daily, along with folic acid (5 mg/day) and comprehensive vitamin-B-complex supplementation. Because of severe anemia (hemoglobin 45 g/L), she received two units of leukocyte-depleted red blood cells, which were well tolerated. Electrolyte correction was achieved with intravenous calcium gluconate, magnesium sulfate, and potassium phosphate. Nutritional rehabilitation was initiated cautiously under dietitian supervision to avoid refeeding syndrome, starting with semi-liquid enteral formulas and gradually advancing to full-calorie intake. The patient was managed using a structured refeeding prevention protocol aligned with international consensus guidelines. Caloric intake was initially limited to approximately 5-10 kcal/kg/day (20-30% of daily needs) and increased slowly over 4-7 days under close clinical and biochemical monitoring. Oral supplementation of phosphate, potassium, and magnesium was provided prophylactically to maintain serum levels in the upper-normal range. As caloric intake increased, thiamine dosing was escalated to 500 mg intravenously two to three times daily before carbohydrate reintroduction. Careful fluid balance management helped prevent sodium-water retention. These combined measures successfully prevented the onset of refeeding syndrome, and the patient remained hemodynamically and biochemically stable throughout the nutritional repletion process. Multidisciplinary care involved specialists from neurology, nutrition, hepatology, and rehabilitation.

Over the following week, her appetite and energy progressively improved, and her weight stabilized. The fever recorded at admission subsided by the third hospital day. Repeat laboratory results demonstrated a rise in hemoglobin to 74 g/L, improvement in serum proteins, and normalization of electrolytes. Her mental status became brighter and more responsive. On neurological reassessment, horizontal nystagmus decreased in frequency, apathy resolved, and muscle strength improved to grade 4/5 in all extremities, although mild gaze limitation persisted. The patient remained hemodynamically stable and free of new deficits.

Psychiatric evaluation during hospitalization confirmed that the patient’s eating restriction stemmed from a subclinical belief of being overweight, although she did not meet the full criteria for an eating disorder. No antipsychotic medication was administered. Environmental control, emotional support, and close nurse observation were prioritized. A multidisciplinary care team-including neurology, psychiatry, nursing, and nutrition-conducted daily assessments. As her thiamine deficiency and cerebral metabolism improved, neuropsychiatric symptoms resolved fully without psychotropic intervention. An outpatient psychiatric referral was arranged upon discharge.

She was discharged on October 20, 2025, in a stable condition with recommendations for a high-protein, high-calorie diet and continuation of oral thiamine (20 mg three times daily), folic acid (0.5 mg three times daily), mecobalamin (0.5 mg daily), polysaccharide-iron complex (150 mg daily), and multivitamin B complex. She was advised to attend regular follow-ups with neurology, hepatology, and nutrition clinics for continued monitoring of metabolic recovery and nutritional status.

## Discussion

WE is a treatable but frequently underdiagnosed neurological emergency resulting from acute thiamine deficiency. While traditionally associated with chronic alcoholism, non-alcoholic forms, particularly those with atypical or incomplete clinical presentations, are increasingly recognized and may account for nearly half of all reported cases. The present case illustrates a rare and preventable occurrence of non-alcoholic WE in a young woman whose self-imposed dietary restriction, driven by distorted body image, culminated in severe malnutrition, electrolyte imbalance, and life-threatening vitamin deficiency. Her presentation underscores the expanding clinical and demographic spectrum of WE beyond alcohol misuse and highlights the diagnostic challenges and vulnerability of young individuals engaging in restrictive eating behaviors.

Thiamine, a water-soluble vitamin obtained primarily through diet, functions as an essential coenzyme for pyruvate dehydrogenase, α-ketoglutarate dehydrogenase, and transketolase, key enzymes in cerebral glucose metabolism and the pentose phosphate pathway. Its deficiency rapidly disrupts ATP production, leading to oxidative stress, neuronal swelling, and selective necrosis in regions with high metabolic demand, such as the periventricular gray matter, thalamus, and mammillary bodies. These metabolic derangements explain the characteristic neuroimaging findings of bilateral symmetric hyperintensities on T2-weighted and FLAIR sequences, as observed in this patient. MRI remains the most sensitive diagnostic tool, particularly in non-alcoholic WE, where clinical manifestations may be incomplete or atypical.

In the present case, the patient exhibited ophthalmoplegia, nystagmus, and mild apathy without overt confusion or ataxia. This partial triad presentation is consistent with reports indicating that only 16-20% of patients demonstrate the full classic triad [5]. The absence of confusion may reflect early detection before the onset of global encephalopathy. In addition, her profound electrolyte abnormalities, hypophosphatemia, hypomagnesemia, and hypocalcemia, likely reflected the combined effects of starvation, poor nutritional intake, and the early stages of refeeding syndrome. Hypomagnesemia, in particular, can impair the phosphorylation of thiamine to its active form, thiamine pyrophosphate, further exacerbating neurological dysfunction despite supplementation [6].

Another remarkable aspect of this case is the coexisting hepatic steatosis and hypoproteinemia, both of which are metabolic consequences of prolonged caloric and protein deprivation. Fatty liver, often seen in eating disorders, reflects impaired hepatic β-oxidation and lipoprotein synthesis. This metabolic profile, combined with low folate and vitamin D levels, provides a biochemical signature of chronic malnutrition rather than a single acute event. The transient fever and elevated liver enzymes observed on admission were likely secondary to hepatic stress and post-transfusion hemolytic turnover, though the fever had already begun to resolve before transfusion was administered.

While WE associated with anorexia nervosa has been described previously, this patient’s presentation is noteworthy for the absence of a formal psychiatric diagnosis. Her restrictive behavior was rooted in a persistent but subclinical belief of being overweight, an increasingly common phenomenon among young adults exposed to unrealistic body-image standards [7]. This observation reinforces the need for early nutritional screening and mental-health evaluation even in individuals who do not meet the strict diagnostic criteria for eating disorders. Clinicians should maintain a high index of suspicion for thiamine deficiency in any patient presenting with unexplained ocular motor dysfunction, weakness, or malnutrition, irrespective of alcohol history.

Prompt recognition and immediate administration of parenteral thiamine remain the cornerstone of therapy. Oral supplementation is often insufficient in the acute phase due to impaired intestinal absorption and depleted tissue stores. The current patient received 100 mg intramuscular thiamine three times daily, leading to rapid stabilization and gradual neurological recovery. This aligns with current recommendations that high-dose parenteral thiamine be initiated empirically in any at-risk patient before confirmation, as diagnostic delay can result in irreversible neuronal loss or transition to Korsakoff syndrome. The favorable recovery observed here also underscores the importance of a multidisciplinary approach integrating neurology, nutrition, hepatology, and rehabilitation to correct deficiencies and prevent refeeding complications [8].

Differential diagnosis was approached systematically, considering structural, metabolic, and autoimmune encephalopathies. The classic triad of ophthalmoplegia, ataxia, and altered mentation, together with rapid onset and nutritional context, prioritized WE. MRI showed symmetric T2/FLAIR hyperintensities in the medial thalamus and periaqueductal gray matter, distinctive for WE and atypical for stroke or encephalitis. CSF analysis was unremarkable, ruling out infectious etiologies. Autoimmune markers and metabolic panels excluded autoimmune encephalitis and hepatic or uremic encephalopathy. Most importantly, the patient's rapid response to parenteral thiamine strongly reinforced the diagnosis and helped exclude mimics.

From a broader perspective, this case highlights an emerging intersection between nutritional neuroscience and societal health behavior. In the era of social media-driven body ideals, voluntary starvation and “clean-eating” trends (voluntary caloric restriction or ‘wholistic’ dietary patterns that avoid grains and carbohydrates) may unintentionally lead to micronutrient depletion, including thiamine. WE in non-alcoholic, young individuals may therefore represent a growing but under-recognized public-health challenge. Early education about balanced nutrition, routine screening for vitamin deficiencies in patients with rapid weight loss, and preventive thiamine supplementation in high-risk groups are essential strategies to avert such outcomes [9].

In summary, this case reinforces that WE should not be confined to the realm of alcoholism but must be recognized as a potential complication of self-imposed malnutrition. The coexistence of severe anemia, electrolyte derangements, and hepatic steatosis reflected a systemic metabolic collapse triggered by chronic dietary restriction. The dramatic improvement following thiamine administration and nutritional rehabilitation emphasizes the reversibility of this condition when promptly diagnosed and treated. Greater clinical vigilance and preventive public-health measures are warranted to protect vulnerable young populations from the neurological consequences of misguided weight-control behaviors.

## Conclusions

This case highlights that WE can result from severe nutritional deprivation unrelated to alcohol use. In this young woman, self-imposed starvation driven by distorted body image led to profound malnutrition, thiamine deficiency, and classical radiological findings of WE. Her presentation, lacking overt confusion but showing ophthalmoplegia and limb weakness, reflects how subtly the syndrome may manifest in non-alcoholic individuals. Early recognition, prompt parenteral thiamine, and nutritional rehabilitation led to notable clinical improvement.

The coexistence of anemia, hepatic steatosis, and electrolyte disturbances illustrates the systemic impact of chronic undernutrition. This case bridges neurology, nutrition, and behavioral medicine, emphasizing that metabolic disease may arise from psychological misperception rather than organic pathology. It serves as a warning against the growing trend of self-restrictive eating among youth and underscores the need for early multidisciplinary intervention.
